# How *Legionella* defend their turf

**DOI:** 10.7554/eLife.48695

**Published:** 2019-06-28

**Authors:** Elisa D Hughes, Michele S Swanson

**Affiliations:** Department of Microbiology and ImmunologyUniversity of Michigan Medical SchoolAnn ArborUnited States

**Keywords:** *Legionella pneumophila*, *Legionella micdadei*, homogentisic acid, pyomelanin, inter-bacterial competition, antimicrobial, Other

## Abstract

Communities of bacteria that cause Legionnaires' disease repel other bacteria by secreting an acid called HGA

**Related research article** Levin TC, Goldspiel BP, Malik HS. 2019. Density-dependent resistance protects *Legionella pneumophila* from its own antimicrobial metabolite, HGA. *eLife* **8**:e46086. doi: 10.7554/eLife.46086

*Legionella pneumophila* is a water-borne bacterium that takes up residence in engineered water systems, and if inhaled by susceptible people, it can cause a severe form of pneumonia known as Legionnaires’ disease. According to the Centers for Disease Control and Prevention, *L. pneumophila* infections are on the rise and are now the leading cause of water-associated illness in the United States. However, a major problem is that decontamination methods often fail to eradicate every single bacterium, which allows populations of bacteria to build up again (Cervero-Aragó et al., 2015; Berjeaud et al., 2016).

One way that *Legionella* manages to persist in the environment is by associating with biofilms – microbial communities that attach to surfaces and encase themselves in a protective matrix (Abu Khweek and Amer, 2018). The microbes in these communities engage in behaviors that mutually benefit each other, such as forming food chains or making up the sugar chains of the biofilm’s protective shield (Elias and Banin, 2012). Inhabitants of the biofilm must also repel other microbes that are unlikely to contribute to the community (Drescher et al., 2014; Özkaya et al., 2017). However, despite these biofilms being a threatening pool of infectious bacteria, little is known about how *L. pneumophila* socially behave. Now, in eLife, Tera Levin, Brian Goldspiel and Harmit Malik from the Fred Hutchinson Cancer Research Center report how highly dense populations of *L. pneumophila* can inhibit the growth of other bacteria belonging to the same or related species of *Legionella* (Levin et al., 2019).

Levin et al. found that *L. pneumophila* secrete a molecule called homogenetisic acid, or HGA for short, which is produced by the amino acids tyrosine and phenylalanine as they convert into more complex molecules. *Legionella* primarily secretes HGA after the bacteria have stopped replicating, during a period known as the stationary phase. Once secreted, HGA combines with oxygen and self-assembles into long chains to form a dark brown pigment. This pigment, called pyomelanin, is known to protect *Legionella* against light damage and to help them acquire iron, which is an essential micronutrient (Steinert et al., 1995; Zheng et al., 2013). Levin et al. show that HGA also has toxic properties that can defend *Legionella* communities from invading microbes.

These properties became evident when the researchers discovered that *L. pneumophila* mutants that fail to inhibit the growth of other *Legionella* bacteria did not produce HGA. Interestingly, secreted HGA is only toxic if oxygen is present in the environment, and Levin et al. argue that a reactive intermediate formed during the conversion of HGA to pyomelanin likely accounts for its inhibitory properties. HGA is also detoxified by reducing agents, including the amino acid cysteine, which must be present for *L. pneumophila* to grow in laboratory cultures.

One conundrum is how *L. pneumophila* avoids the toxic effects of HGA. As secretion of the inhibitor increases when the bacteria are in the stationary phase – a state typically associated with high bacterial density – Levin et al. speculated that genes involved in either quorum sensing (which leads to changes in gene regulation based on population density) or the stringent stress response may promote resistance to HGA. However, deleting known genes in each of these widespread cell-signaling pathways does not alter resistance or susceptibility to HGA. Instead, *L. pneumophila* cells are only susceptible to inhibition when at low density, regardless of whether they are in the stationary or replicating phase. It therefore remains to be discovered how individual *L. pneumophila* cells at high density protect themselves from HGA.

Thinking about the natural habitats of *L. pneumophila*, Levin et al. envision individual bacterial cells sticking to a surface, such as the interior of a water pipe (Figure 1). As *L. pneumophila* begin to replicate, they form a small colony, then grow to form a large cooperative community, likely sharing resources liberated by digestive enzymes that they have secreted (White and Cianciotto, 2019). After reaching a certain density, the residents protect their turf from other bacteria by releasing a pulse of HGA. Since the high concentration of bacteria in the colony also triggers resistance to HGA, the community is automatically protected from the inhibitor.

**Figure 1.**
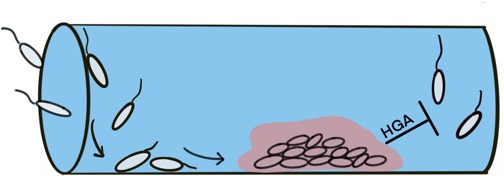
High-density colonies of *Legionella* protect themselves by secreting pulses of HGA. The development of *Legionella* colonies begins with individual cells adhering to a surface, such as the inside of a water pipe. After reaching a certain density, the bacteria intensify the secretion of HGA, which prevents other *Legionella* cells from joining the colony. Image credit: Elisa D. Hughes (CC BY 4.0).

The work of Levin, Goldspiel and Malik provides an important insight into how *Legionella* persist within microbial communities, and also identifies several questions that warrant further investigation. First, as biofilms comprised of a single species are not common in nature (Elias and Banin, 2012), it would be useful to know more about the impact of HGA on non-*Legionella* bacteria. In addition, understanding how the reactive intermediate of HGA is able to inhibit *Legionella*, and how individuals within dense colonies of the pathogen acquire resistance, could significantly advance the broad field of microbiology ecology. Finally, it is tempting to speculate that the HGA inhibition and resistance pathways discovered by Levin et al. could help identify new ways of eliminating pathogenic *Legionella* from engineered water systems.
